# Thickness Characterization of Steel Plate Coating Materials with Terahertz Time-Domain Reflection Spectroscopy Based on BP Neural Network

**DOI:** 10.3390/s24154992

**Published:** 2024-08-01

**Authors:** Xuelei Jiang, Ying Xu, Hang Hu

**Affiliations:** 1 School of Civil and Environmental Engineering, Harbin Institute of Technology, Shenzhen 518055, China; jiangxl_szu@163.com (X.J.);; 2 Guangdong Provincial Key Laboratory of Intelligent and Resilient Structures for Civil Engineering, Harbin Institute of Technology, Shenzhen 518055, China

**Keywords:** terahertz time-domain reflection spectroscopy, BP neural network, coating materials, coating thickness, defect identification

## Abstract

Accurate monitoring of steel plate coating thickness is crucial in construction quality control and durability assessments. To address this challenge, this study introduces a terahertz time-domain reflection spectroscopy based on a BP neural network model to achieve a quantitative visualization characterization of coating thickness. The BP neural network eliminates the inherent dependence of terahertz reflection spectroscopy on the refractive index value in thickness calculation. This trained BP neural network model effectively establishes a functional relationship between signal feature parameters and the corresponding thickness values. Additionally, the proposed model can innovatively measure different coating materials’ refractive indexes, revealing the corresponding values for the black paint, white paint, epoxy resin, and rubber as 2.212, 1.967, 1.924, and 2.185, respectively. The experimental results demonstrate the trained BP neural network model possesses remarkable accuracy in predicting coating thickness within the scanning area, achieving a precision level exceeding 96%. This method enables the visualization of coating thickness and the extraction of thickness characterization values. Furthermore, using the thickness imaging results as a reference, the method can accurately identify the thickness abnormalities across the scanning area, locating the position and size of potential defects such as internal scratches and foreign object defects. This innovative approach offers a superior means of monitoring and assessing the thickness distribution quality of the steel plate coating layer materials.

## 1. Introduction

Steel plate materials have been widely used in civil engineering owing to their lightweight, high-strength, and good toughness characteristics. However, due to the unstable chemical properties of steel plate materials, covering and protecting their surface in practical engineering is critical, as it improves the durability of steel plate structures [1]. Therefore, monitoring the thickness of the surface coating layer is important for evaluating steel plate structure construction quality and structural durability. Commonly used methods include the probe method and dry paint film thickness gauge. The former is a lossy testing method, which damages the coating, while the latter, although a non-destructive testing method, measures single-point thickness and cannot evaluate the overall thickness distribution or identify internal defects. Furthermore, some scholars have estimated several non-destructive testing methods, such as ultrasonic testing [2], eddy current testing [3], and thermal imaging [4], for the thickness detection of steel plate coating materials. Although these non-destructive testing methods can evaluate the thickness of the coating materials by monitoring relevant physical quantities, they still have inherent limitations. For instance, ultrasonic detection technology is inadequate for thickness distribution detection and cannot conduct online monitoring. Eddy current testing is appropriate for conductive materials with shallow surfaces but cannot easily detect deeper defects. Thermal imaging technology is also affected by foreign bodies or material defects and is unsuitable for quantitative thickness characterization. In summary, those methods are susceptible to various factors such as the temperature, surface flatness of the coating layer, or internal defects, making it challenging to characterize the thickness of the coating material quantitatively.

With the development of ultrafast femtosecond lasers and terahertz emitting and receiving device technology, terahertz time-domain spectroscopy has been widely employed in various fields such as aerospace [5], terahertz communication [6], environmental monitoring [7], and material characterization [8]. Terahertz imaging characterization methods are based on the extraction of feature parameters (such as the time-domain amplitude, frequency-domain amplitude, maximum peak value of the time-domain signal, minimum peak value of the time-domain signal, and peak-to-peak value) from terahertz time-domain reflection signals at all point positions in the scanning area [9,10]. Therefore, some scholars have used this terahertz technology in civil engineering to explain the non-destructive imaging characterization of steel plate coating materials. For instance, Tu et al. [11] extracted the feature values from the terahertz pulsed signals of marine coatings using the Hilbert transform (HT) and wavelet transform modulus maximum (WTMM). This method can accurately locate the coating defects. Zhao et al. [12] proposed a method to obtain the thickness and refractive index of the thermally grown oxide under thermal barrier coatings. The thickness accuracy of the thermally grown oxide layer was verified by SEM testing, proving this method has a reasonable agreement. Tao et al. [13] investigated the application of terahertz spectroscopy and imaging in non-contact, non-destructive testing methods in various industrial sectors. They concluded that the terahertz method can visualize materials in civil engineering, such as concrete, insulating foam, plastics, natural gas pipelines, electrical cables, and thermal barrier coatings. These methods exhibit a pronounced dependence on the relative magnitude parameter values, thereby limiting their ability to quantitatively characterize the thickness of the coating material.

According to the transmission mechanism of the terahertz time-domain spectroscopy signal, the thickness of coating layer materials can be accurately detected. Specifically, the reflected terahertz signals are generated at the upper and lower interfaces of the steel plate coating layer. By extracting the delay time difference corresponding to the amplitudes of the reflected wave signal, the thickness of the coating layer can be calculated based on the simplified thickness formula [14] $d=\frac{c\Delta t}{2\bar{n}}$, where *d* is the thickness of the coating layer, Δ*t* is the delay time difference parameter, *c* is the speed of light, and $\bar{n}$ is the refractive index value. This formula has been used to measure the thickness of the coating layer or/and composite layer materials. Additionally, Liu et al. [15] employed the terahertz time-domain spectroscopy to measure the thickness distribution of the thermal barrier coatings and demonstrated that the measurement deviation of this method was within 12.1 μm by comparing it with the metallography results. Jiang et al. [16] extracted the delay time difference in the reflected terahertz signal to detect the coating and corrosion layer thicknesses. This scheme visualizes the thicknesses in a three-dimensional appearance and provides a new way to quantify the corrosion. Kumar et al. [17] utilized terahertz imaging in reflection mode to measure thickness and distinguish the healthy and deboned/inclusion areas of a cork phenolic-composite structure. Liu et al. [18] used terahertz technology to process and image the internal defects within complex structures. It can detect all pore, debonding, and crack defects, with a minimum size of 3 mm for pores and debonding and a minimum thickness of 1 mm for cracks. The experimental results show this technology has the potential for detecting internal defects and performing accurate dimensional measurements in complex structures. Although the simplified thickness formula is highly accurate, it mainly depends on the refractive index of the measured materials. Since the refractive index is an intrinsic property of the material, influenced by various factors such as composition, structure, and density, it can be measured using terahertz time-domain transmission spectroscopy [19]. However, this measurement process requires scraping off the coating materials from the surface of steel plates and ensuring the accurate sample thickness measurement and surface roughness. 

In characterizing the thickness distribution of coating materials using terahertz spectroscopy technology, a large number of datasets need to be processed. The propagation and absorption characteristics of terahertz waves by different coating materials are jointly influenced by material composition, additives, and coating process conditions. Each coating material has a characteristic terahertz spectral signal data. Thus, this paper proposes a functional relationship between the terahertz time-domain reflection spectrum signal and the corresponding thickness value. Specifically, the developed scheme bypasses the requirement to measure the refractive index of the material, enabling direct data processing and thickness characterization. In theory, BP neural networks can effectively fit any rational function [20] and therefore, some scholars have utilized the computational power of BP neural networks to conduct relevant fitting explorations. For instance, Tu et al. [21] utilized a neural network-based hybrid signal procession method to enhance the resolving capability of terahertz pulsed imaging to the thin coating layer. The various features in terahertz signals were obtained through Fourier deconvolution, fast Fourier transform, and wavelet analysis and were used as the input vectors for the neural network. Liu et al. [22] explored the innovative application of the terahertz technique combined with a machine learning approach to predict the porosity of the thermal barrier coatings. Meanwhile, some scholars [23,24,25] have proven that the BP neural network algorithm can fit the functional relationship between feature parameters and corresponding target values, with high applicability and accuracy. 

This study proposes a BP neural network training model that establishes the functional relationship between feature parameters in terahertz time-domain reflection signals and thickness values, incorporating different coating materials. Specifically, the terahertz signal and true thickness value are acquired using a terahertz time-domain reflection spectrometer and a coating thickness gauge, and are utilized as input layer parameters and the desired output parameter, respectively, in the BP neural network model. Then, through extensive training with the BP neural network model, we achieve an optimal thickness fitting relationship to employ for the thickness imaging characterization of steel plate samples. Finally, the defects in the coating layer are identified by considering thickness imaging results as the standard. Using this training model, batch data processing of terahertz time-domain reflection spectroscopy signals, thickness characterization, and defect identification of steel plate samples with various coating layers are achieved. This solution provides a non-destructive testing method for evaluating the thickness and identifying the defects in the coating layer materials.

## 2. Materials and Methods

### 2.1. Experiments Setup

**Figure 1:**
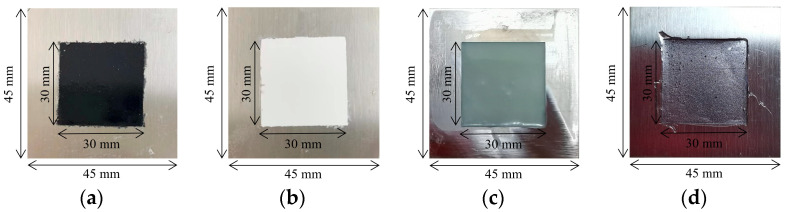
Steel plate samples for BP neural network training. Coating materials include (**a**) black paint; (**b**) white paint; (**c**) epoxy resin; (**d**) rubber.

Figure 1 depicts the steel plate samples with different coating materials, which were prepared to train the BP neural network model. The coating materials included black paint, white paint, epoxy resin, and rubber. The size of the Q235 steel plate sample was 45 mm×45 mm. The black and white paints were provided by Guangdong Haoshun ODIS Technology Co., Ltd. (Zhaoqing, China), and the paint was evenly sprayed on the surface of the steel plate three times every two days. The prepared samples are depicted in Figure 1a,b. Furthermore, epoxy resin and rubber materials were provided by Hunan Baxiongdi New Materials Co., Ltd. (Changsha, China) and Shenzhen Ausbon Co., Ltd. (Shenzhen, China), respectively. Additionally, a stainless-steel template was employed to prepare the steel plate coating samples with coating materials of epoxy resin and rubber, occupying an area of 30 mm×30 mm and a thickness of about 1 mm (Figure 1c,d). The steel plate coating area was divided into measurement point positions, with points in the *x* and *y* directions set to $\left(x,y\right)=(30,30)$ at an interval of 1 mm. At the measurement area, a terahertz time-domain reflection spectrometer and a coating thickness gauge were used to obtain the terahertz time-domain reflection signal and thickness value at the corresponding positions, respectively. The model of this coating thickness gauge instrument is DR5000S, with a measurement range of 0–5000 μm, a measurement accuracy of ±3 μm, and a resolution of 0.1 mm.

**Figure 2:**
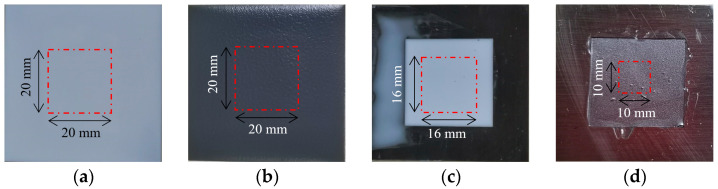
Steel plate samples for thickness measurement. Coating materials include (**a**) white paint; (**b**) white–black paint; (**c**) epoxy resin; (**d**) rubber.

The coating material’s BP neural network training model can process the terahertz time-domain spectral data to characterize coating thickness in the scanning area. Figure 2 illustrates the prepared steel plate samples with different coating materials, where Figure 2a presents a steel plate sample coated with white paint uniformly sprayed onto the steel plate surface. Additionally, by further spraying black paint on the white paint as the steel plate primer, the white + black paint steel plate sample was prepared (Figure 2b). Regarding the steel plate samples presented in Figure 2a,b, the scanning area was 20 mm×20 mm, with a scanning interval of 0.16 mm, a scanning number of 125×125, and a total of 15,625 scanning points. The scanning parameters for the epoxy resin and rubber steel plate samples were also defined. The scanning area for the epoxy resin steel plate sample was 16 mm×16 mm with a scanning number of 100×100 for 10,000 scanning points. The scanning area for the rubber steel plate sample was 10 mm × 10 mm, and the scanning number was 62×62=3844.

**Figure 3:**
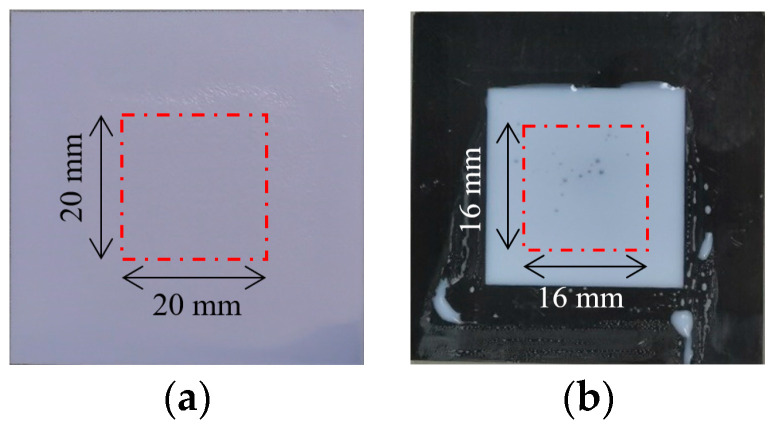
Defective coating material samples. (**a**) White paint sample with scratch defect. (**b**) Epoxy resin sample with foreign object defect.

Based on the coating thickness characterization, the internal defects within the coating material were identified. Figure 3a presents the white paint steel plate sample with scratch defects. During the spray preparation of the white paint sample, the middle area was intentionally scratched and further covered with white paint. Figure 3b depicts the epoxy resin steel plate sample with foreign object defects. During sample preparation, a rectangular paper foreign object (4 mm×2.5 mm size) was initially placed on the surface of the steel plate, followed by covering with epoxy resin. The scanning area for the paint and epoxy resin samples was set to 20 mm×20 mm and 16 mm×16 mm, with a total number of scanning points of 125×125=15,625 and 100×100=10,000, respectively.

A Terapulse 4000 experimental setup, produced by TeraView Co., Ltd. (Cambridge, UK) and supplied by Shenzhen Institute of Terahertz Technology and Innovation, Shenzhen, China, was used. This system is mainly composed of ultrashort pulse laser, terahertz emitter, terahertz detector, and time delay device. The emitter and detector are both photoconductive antennas (PCA), with the substrate being low-temperature-grown gallium arsenide (LT-GaAs). In terahertz system, the ultrashort pulse laser produces a femtosecond fiber laser pulse with a center wavelength of 780 nm. Then, it is divided into pump beam and probe beam by the beam splitter. When the pump beam impacts the emitter, a pulse terahertz wave is produced. After penetrating the sample, the waves will carry the optical information with the help of the parabolic mirror. The time delay device causes an optical time delay of the probe beam by parallel translation; then, the probe beam and the terahertz waves are simultaneously focused on the detector, allowing for the whole terahertz time-domain signal to be collected. The parameters of Terapulse 4000 were as follows. The spectral range was between 0.06 and 4.00 THz, the spectral signal-to-noise ratio was larger than 65 dB, and the spectral resolution was 1.2 cm^−1^. In terms of scanning imaging capabilities, this setup provided flexibility in selecting scanning locations and areas within the structure through single-point measurements across the scanning area. The diameter of the laser spot used for measurement was approximately 2 mm, while the resolution of terahertz imaging can reach up to 0.07 mm. Additionally, the scanning parameters encompassed a scanning range covering the *x* and *y* directions from 0 to 50 mm, with a scanning step size ranging from 0.08 to 1 mm (the step size must be a multiple of 0.04), and waveform rates of fast (15 Hz), medium (8 Hz), or slow (3 Hz). More detailed information was presented in the literature [26].

### 2.2. BP Neural Network Theory

**Figure 4:**
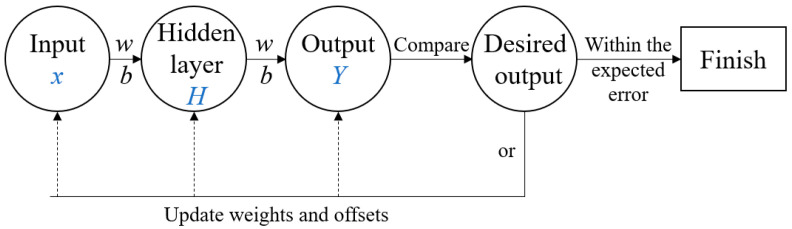
Schematic diagram of BP neural network.

In order to image the coating thickness of steel plate and bypass the intricate measurement of refractive index value in conventional formulas, this study trains and fits the functional relationship between the feature values of terahertz time-domain spectral signal and thickness using a BP neural network. By employing this trained model, processing the signal data of terahertz time-domain spectra directly is feasible, enabling the thickness characterization and defect identification of the coating materials. The main feature of the BP neural network is propagating signals forward while errors are relayed backward. Figure 4 illustrates the algorithm’s schematic. By constantly adjusting the network’s weight w and threshold b, the network error is reduced to below the expected error. Therefore, this study extracted the feature and corresponding thickness values of the coating layer, and then trained the BP neural network model. 

**Figure 5:**
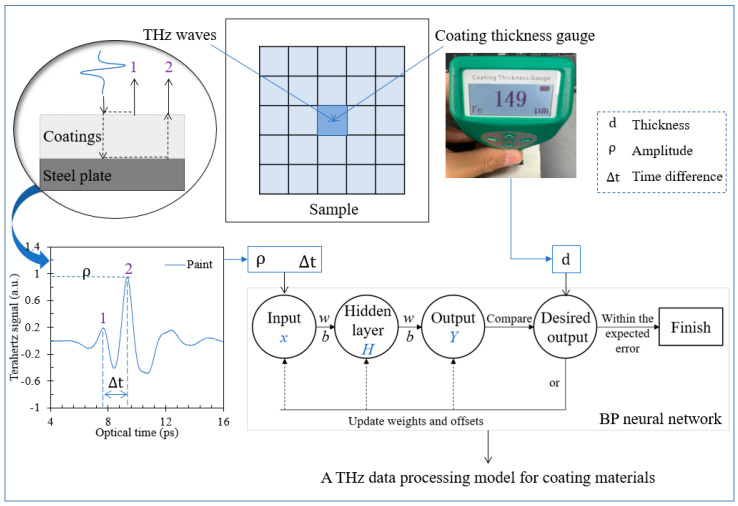
Establishment of THz data processing model for coating materials based on BP neural network.

Figure 5 depicts the application principle of the data processing model for the terahertz signal of different coating materials. The terahertz time-domain reflection signal and thickness value are obtained at each measurement point using the spectroscopy instrument and coating thickness gauge, respectively. This means the two methods correspond one-to-one at the measurement position of each point, and the measured thickness value data are used as the expected output in the terahertz data processing model. As depicted in Figure 5, the electromagnetic wave experiences both reflection and transmission when it reaches the surface interface of the upper surface of the coating layer. The reflected electromagnetic wave forms the signal amplitude 1 in the time-domain spectrum, while the transmitted electromagnetic wave reflects and forms the signal amplitude 2 when it penetrates the coating layer to reach the lower surface, the interface between the cover layer and the steel plate. In theory, when the thickness of the coating layer is thin, amplitude swallowing phenomenon will occur. Meanwhile, as the thickness gradually increases, signal amplitude 2 will experience intensity dispersion attenuation until it weakens below the noise intensity. Based on this, the thickness measurement range of different coating layer materials can be obtained by terahertz reflection spectral signal. The difference in delay time between amplitudes 1 and 2 is linked to the refractive index value and thickness of the coating material. Therefore, this work exploits as input layer parameters the signal amplitude ρ and the delay time difference Δt in the terahertz time-domain spectroscopy signal. Meanwhile, the thickness of the coating layer is the desired output parameter in the BP neural network. Besides, the measurement data of each coating material sample is divided into a training set and a test set (8:2 ratio), providing 720 training and 180 test sets. 

## 3. Construction of BP Neural Network

This study employs the feature values extracted from terahertz time-domain spectroscopy as input parameters, where the thickness value serves as the output parameter for training the BP neural network model. The input parameters involve the signal amplitude ρ and the delay time difference Δt. Using supervised learning, the correlation between the weight of each layer and the expected thickness value is established by continuously adjusting the weight of neurons in the hidden layers to enhance the accuracy of the predicted thickness output. Hence, this trained model was applied to process terahertz time-domain signal data and facilitated the characterization of the thickness of coating materials. 

### 3.1. Data Preprocessing

**Figure 6:**
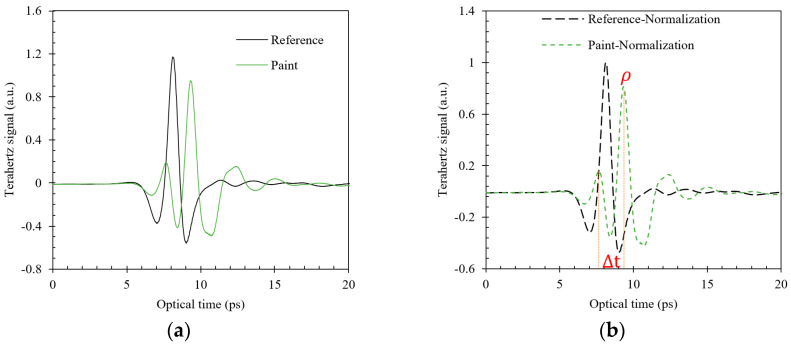
Normalized processing results of THz signal of paint layer. (**a**) Original signals. (**b**) Normalized signals.

The amplitude of the received terahertz time-domain signal influences the external temperature, humidity, coating type, and terahertz instrument. Hence, the data must be normalized before training the neural networks to eliminate the influence of data units on the training accuracy of neural networks and ensure an appealing training time and convergence speed. Thus, the terahertz reference signal dataset is remapped to the [–1, 1] range, and then terahertz sample signals of different coating materials are scaled down proportionally. This work relies on the fuzzy variable method to normalize the dataset. Precisely, the terahertz time-domain signal is preprocessed using the logistic transformation. Then, the dataset is normalized using fuzzy quantization, as presented below:

(1) $$y^{\prime }=\frac{1}{1+e^{-y}}$$

(2) $$y^{\prime \prime }=\frac{1}{2}+\frac{1}{2}[\frac{\pi }{T_{max}-T_{min}}\times (x-\frac{T_{max}-T_{min}}{2})]$$

where y is the sample value, y′ is the pretreatment sample value, y″ is the normalized sample value, $T_{max}$ is the maximum value in the sample, and $T_{min}$ is the minimum value in the sample. Considering the paint coating sample as an example, Figure 6 presents the terahertz reflection signal results before and after normalization. The amplitude of the reference signal is derived from the reflection of the surface of the steel plate. For the coating material samples, the amplitude of the first signal presents a minor difference associated with the surface flatness of the coating material. When the terahertz signal passes through the coating material medium, the attenuation degree of signal amplitude bears a direct non-linear relationship with the thickness of the material, and the relative magnitude of the amplitudes 1 and 2 in the time-domain spectrum are uncertain. Therefore, the reference and sample signals are initially normalized during data processing. Subsequently, the sample signal’s second amplitude is chosen as an input parameter for the BP neural network model. Finally, the delay time difference is extracted from the terahertz time-domain signal. 

**Figure 7:**
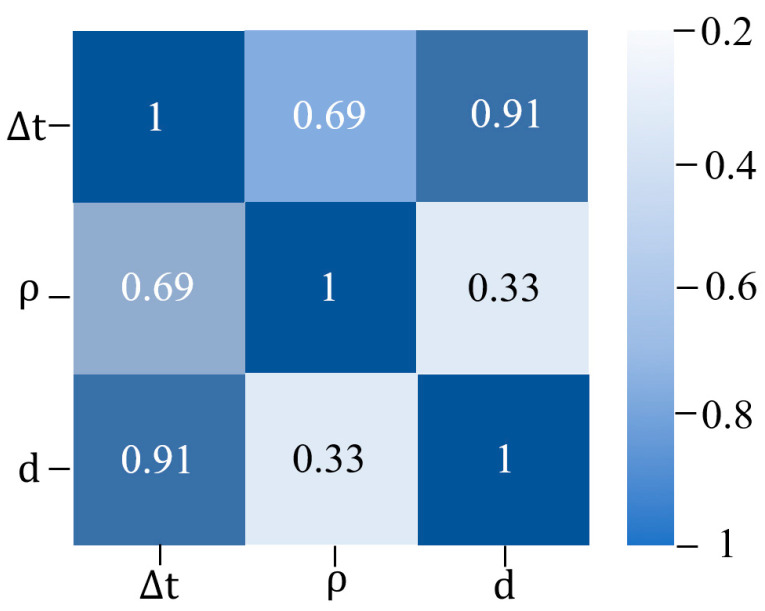
The correlation coefficient relationship diagram of the parameters in BP neural network model.

The correlation analysis serves as a validation tool for theoretical models or experimental setups. Therefore, in this study, the correlation coefficient relationship between parameters in the input layer and the output layer is revealed using Python 3.11. As shown in Figure 7, this correlation is established through the associated color (white to blue) and the value range (0 to 1) so that the higher the correlation coefficient, the more the color in the graph approaches blue. The color-coded map not only reveals the strength of the correlations but also facilitates comparisons between different parameters. The correlation strength of the input parameters (delay time difference and signal amplitude) on the output parameter (coating thickness) is the particular focus of this study. Regarding the thickness value d, the delay time difference parameter Δt in the terahertz reflection signal holds the highest influence coefficient, which is 0.91. This finding is intimately tied to the fundamental physical principle governing the interplay between the terahertz spectral signal (namely, the delay time difference) and the thickness of the coating material. While the influence coefficient of the signal amplitude ρ is 0.33, it is obviously lower than that of the time difference parameter. It means other factors may play a more prominent role in influencing the output parameter in training the BP neural network model. In summary, a high coefficient is observed between the parameters of the input layer and the output layer, substantiating the rationality and accuracy of the delay time difference parameter Δ*t* and the signal amplitude ρ as the input layer parameters in this study.

### 3.2. BP Neural Network Framework

**Figure 8:**
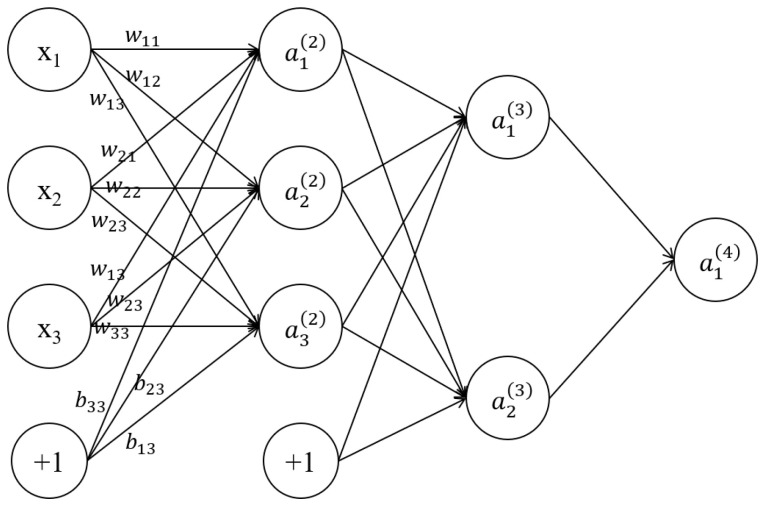
Basic architecture of BP neural network.

This study selects the signal amplitude ρ and the delay time difference Δt as the neural network’s input layer parameters, while the coating layer’s thickness at the corresponding position is the output layer parameter for neural network training. Figure 8 illustrates the BP neural network architecture. $x_{i}$ denotes the three neurons in the input layer, $w_{ij}$ is the weight of the neurons between the two adjacent hidden layers, $b_{ij}$ is bias, and y=f(x) is the activation function. $a_{i}^{j}$ is the *i*-th neuron in the *j*-th layer. The neurons $a_{1}^{\left(2\right)}$, $a_{2}^{\left(2\right)}$, and $a_{3}^{\left(2\right)}$ can be calculated using Formulas (3), (4), and (5), respectively. $h_{w,b}\left(x\right)$ is the calculated value of the third layer neurons $a_{1}^{\left(3\right)}$, as presented in Formula (6). The input layer parameters are weighted and summed and then passed into the hidden layer to express the forward propagation process of the BP neural network and the output thickness result d′.

(3) $$a_{1}^{\left(2\right)}=f(w_{11}^{\left(1\right)}x_{1}+w_{12}^{\left(1\right)}x_{2}+w_{13}^{\left(1\right)}x_{3}+b_{11}^{\left(1\right)})$$

(4) $$a_{2}^{\left(2\right)}=f(w_{21}^{\left(1\right)}x_{1}+w_{22}^{\left(1\right)}x_{2}+w_{23}^{\left(1\right)}x_{3}+b_{12}^{\left(1\right)})$$

(5) $$a_{3}^{\left(2\right)}=f(w_{31}^{\left(1\right)}x_{1}+w_{32}^{\left(1\right)}x_{2}+w_{33}^{\left(1\right)}x_{3}+b_{13}^{\left(1\right)})$$

(6) $$h_{w,b}\left(x\right)=a_{1}^{\left(3\right)}=f(w_{11}^{\left(2\right)}a_{1}+w_{12}^{\left(2\right)}a_{2}+w_{13}^{\left(2\right)}a_{3}+b_{21}^{\left(2\right)})$$

Accurately characterizing the functional relationship using a BP neural network requires generating a reverse data stream by comparing the error of the output result and the expected result (d′ and d) and then adjusting the weights $w_{ij}$ in the backpropagation process. This alternating computation in positive and negative directions realizes the network learning process. The error per sample is:

(7) $$E\left(i\right)=\frac{1}{2}\sum_{k=1}^{n}{\left(d_{k}(i)-y_{k}(i)\right)}^{2}$$

(8) $$w_{ij}^{\left(l\right)}=w_{ij}^{\left(l\right)}-\alpha \frac{\partial E}{{\partial w}_{ij}^{\left(l\right)}}$$

(9) $$b_{i}^{\left(j\right)}=b_{i}^{\left(j\right)}-\alpha \frac{\partial E}{{\partial b}_{i}^{\left(j\right)}}$$

where α is the initial learning rate, $b_{i}^{\left(j\right)}$ is the initial threshold, and $w_{ij}^{\left(l\right)}$ is the weight between different hidden layers. The learning rate in the BP neural network algorithm determines the weight change generated during each training cycle. A small learning rate (0.06) is selected to ensure the system’s stability. The initial weights between the input layer and the hidden layer, the initial threshold of the hidden layer neurons, the initial weights between the hidden layer and the output layer neurons, and the initial threshold of the output layer neurons in the neural network are randomly set between (−1, 1) to improve the expected accuracy of the neural network. Meanwhile, this study employs a three-layer network, and the node numbers of the input layer, hidden layer, and output layer are determined based on the following empirical formula:

(10) $$n<\sqrt{\omega +\sigma }+\lambda$$

where n is the reference value of the node number in the hidden layer, ω is the node number of the input layer in the neural network, σ is the node number of the output layer in the neural network, and λ is a constant between 0 and 10. 

**Figure 9:**
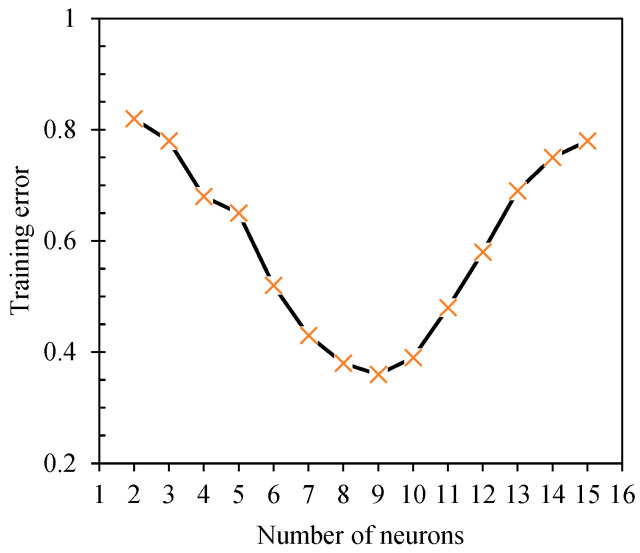
Relationship between the number of neurons and training errors.

Taking the black paint coating sample as an example, the number of nodes in the network’s hidden layer is randomly assigned a value within the 2–12 range. Drawing from the network training results of 2–12 different neurons, Figure 9 summarizes the root mean square error for varying neuron nodes, which presents a decreasing trend, indicating that as the number of neurons increases, the information exchange between neural networks intensifies, boosting the computational accuracy of those networks. However, when the number of neurons exceeds nine, the root-mean-square error increases, and the calculation accuracy decreases. This suggests that a larger number of neurons may cause the neural network to fall into the local optimal “trap”, thereby reducing the network’s generalization ability. Therefore, the number of nodes in the input, hidden, and output layers are set as 3, 9, and 1, respectively. The constructed neural network model has a [3-9-1] structure. 

### 3.3. Network Optimization

**Figure 10:**
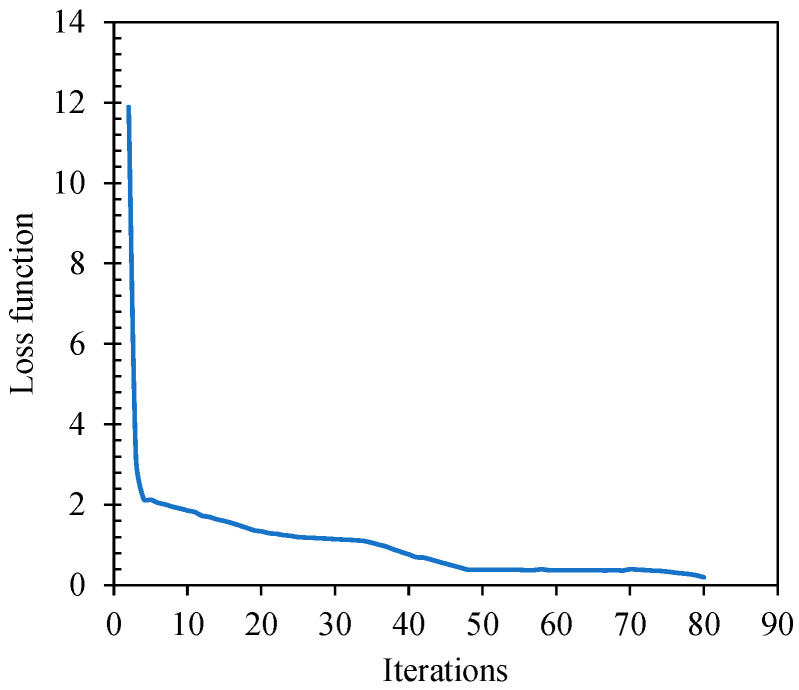
Gradient descent plot.

The BP neural network belongs to the gradient descent algorithm, which essentially aims to guide the model to find the fastest loss function decline direction. This work effectively combines the characteristics of the stochastic gradient algorithm and the RMSprop algorithm, ensuring the weight parameters can be adjusted backward and repeatedly before the gradient function approaches zero. The weight parameter associated with the least error is obtained when the adjustment stops. Additionally, the descending feature of this model is characterized by a smaller step size and a slower rate of descent as it approaches the target value. Thus, this paper determines the fastest decreasing path of the loss function by analyzing its relationship with the number of iteration steps. The loss function reflects the offset distance between the model fit value and the true value, and it can be used to evaluate the fit value. Figure 10 illustrates the changes in the loss function during the training and testing of the black paint layer of the steel plate sample. Figure 10 highlights that the loss function is a non-negative function, and under the influence of the gradient descent algorithm, it continuously decreases and approaches zero. This study defines a minimum loss value of 0.2, controlling the model’s end time and obtaining the optimal solution for training the model. 

## 4. Processing Data Results of the BP Neural Network

**Figure 11:**
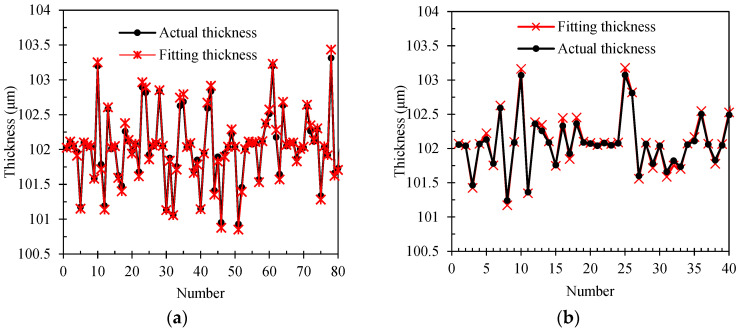
Thickness fitting results of black paint steel plate sample based on BP neural network. (**a**) Training set. (**b**) Test set.

The fitting thicknesses of the training and test set can be determined by training various coating materials’ terahertz time-domain spectral signals. Each steel plate coating sample yields 900 measurement data points, divided into a training set (comprising 720 points) and a test set (comprising 720 points), with an 8:2 ratio. Hence, utilizing the steel plate sample coated with black paint as a case, this research delves into the precision of the predictive model for fitting the thickness of the coating layer. Figure 11 presents a comprehensive comparison between the model-fitting thickness values and their corresponding actual measurements, offering a quantitative assessment of the model’s accuracy. Figure 11a compares the partial model fitting thickness and true thickness of 80 training sets. It can be observed that the accuracy of the fitting and true thickness is higher when the thickness of the black paint coating is around 102 μm. However, when the coating thickness exceeds 102.25 μm, the fitting thickness slightly exceeds the true thickness, while for coating thickness below 101.75 μm, the fitting thickness slightly falls short of the true thickness. Overall, the training set accuracy for the black-paint-coated steel plate sample is over 98.6%. Furthermore, the test set data is processed through the trained model, with Figure 11b highlighting that compared with the true thickness, the test set data has a high fitting thickness accuracy that exceeds 96.5%. The results demonstrate that the functional relationship between the terahertz time-domain reflection spectrum signal and the thickness value of the coating steel plate can be obtained by BP neural network model training, exhibiting a high fitting accuracy.

In this study, the BP neural network model is trained for steel plate samples with white paint, rubber, and epoxy resin coating materials. Table 1 highlights that the training accuracy of BP neural network models exceeds 96%, with 96.5% for black paint, 97.1% for white paint, 97.7% for rubber, and 97.2% for epoxy resin. Additionally, the refractive index of different coating materials could be calculated based on the fitting thickness value and the feature parameter (delay time difference parameter Δt) extracted from the terahertz time-domain spectrum signal. The refractive index values for black paint, white paint, rubber, and epoxy resin are 2.21, 1.97, 2.19, and 1.92, respectively. Compared with the measured values from the terahertz time-domain transmission spectrum in the literature [26], the proposed method exhibits higher accuracy. It provides a new approach for calculating the refractive index of coating materials based on the terahertz time-domain reflection spectrum, while it is not the primary focus of our study. Through the trained BP neural network, a robust functional relationship between the terahertz signals of each coating material and the corresponding thickness values is established. Subsequently, the model is applied to batch process the signals from the coating steel plate samples in the expanded dataset with a high level of prediction accuracy.

## 5. Thickness Imaging of Coating Materials Based on the BP Neural Network

### 5.1. Thickness Characterization of Coating Materials

**Figure 12:**
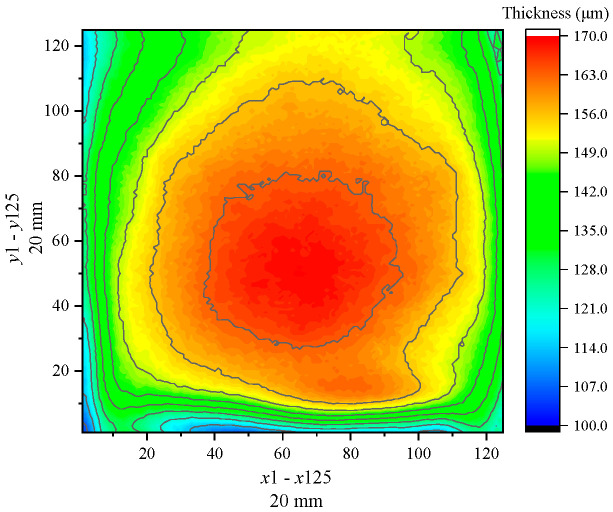
Thickness imaging of white paint sample.

**Figure 13:**
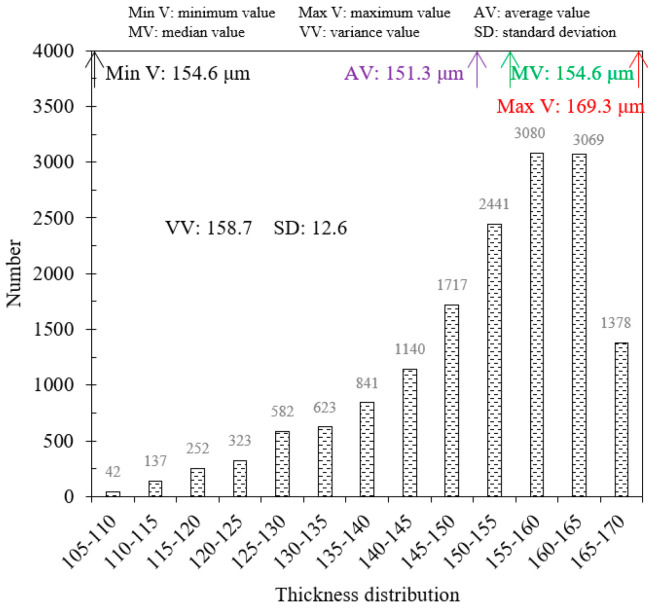
Extraction of the thickness characteristic values in white paint steel plate sample.

The trained BP neural network model can be applied to process the terahertz signal data and characterize the thickness of different coating materials. Considering the white paint steel plate sample as an example, the 2D thickness imaging results in the scanning area are depicted in Figure 12. The scanning area size of the sample is 20 mm×20 mm, with a scanning resolution set to x×y=125×125. The thickness distribution of the white paint layer is highest in the middle and decreases towards the sides. Additionally, the Python algorithm is used for the data analysis of the thickness values, allowing the statistical analysis of different thickness intervals and extraction of thickness characteristic values. Figure 13 infers that the thickness of the white paint layer in the scanning area ranges between 105.24 and 169.29 μm, with an average thickness of 151.3 μm and a median thickness of 154.6 μm. Additionally, within the thickness interval of 5 μm, the highest number of thickness values is observed between 155 and 160 μm, totaling 3080. The range of 150 to 165 μm accounts for 55% of the total number. Furthermore, the variance and standard deviation of the thickness value in the scanning area, which are 158.7 and 12.6, respectively, can be extracted. These values can serve as evaluation indices for assessing the distribution smoothness of the coating layer. 

**Figure 14:**
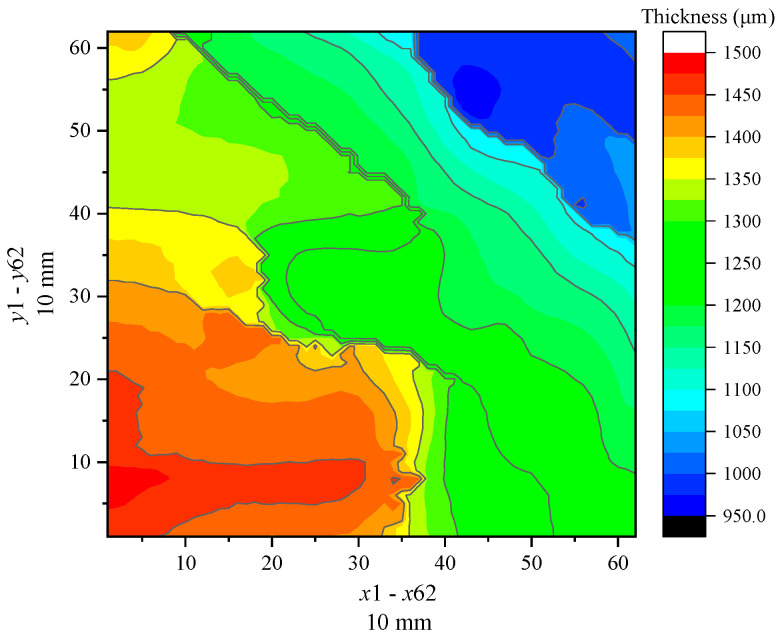
Thickness imaging of rubber sample.

**Figure 15:**
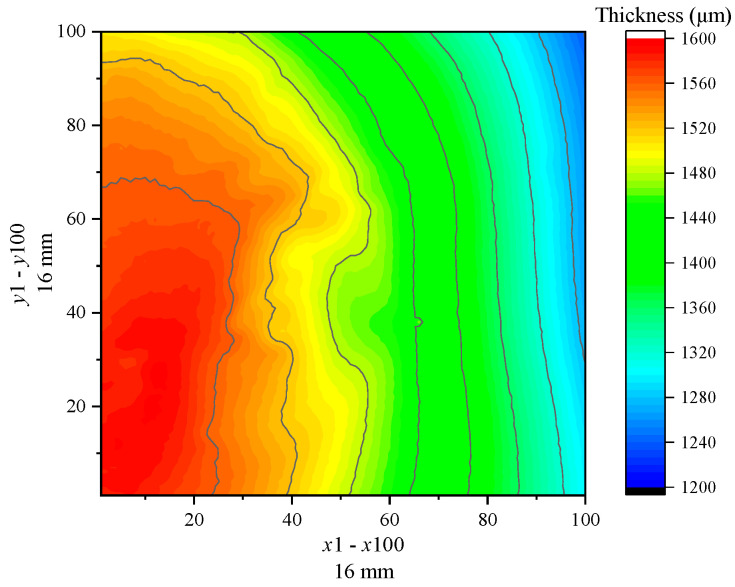
Thickness imaging of epoxy resin sample.

**Figure 16:**
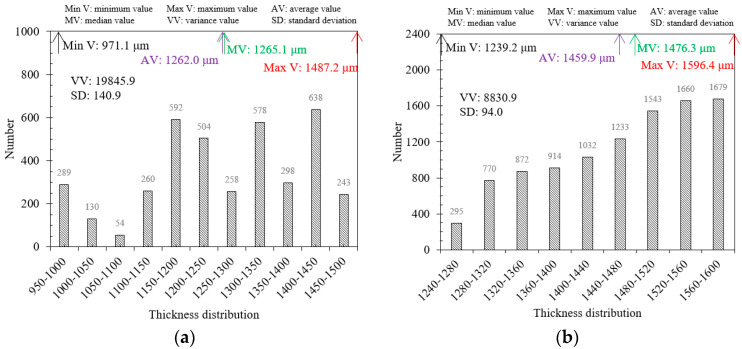
Extraction of the thickness characteristic values in steel plate samples with different coating materials. (**a**) Rubber. (**b**) Epoxy resin.

Similarly, the BP neural network model is employed to process the terahertz signals and thickness characterization of the rubber and epoxy resin coating steel plate samples. Figure 14 and Figure 15 present the thickness imaging results of the samples in the scanning area. The scanning area size of the rubber coating steel plate sample is 10 mm×10 mm, with a scanning point configuration of x×y=62×62, resulting in a total of 3844 scanning points in the scanning area. The scanning area size of the epoxy resin steel plate sample is 16 mm×16 mm, with a scanning point configuration of x×y=100×100, yielding a total of 10,000 points. Figure 14 and Figure 15 compare the imaging results and reveal that the thickness distribution of the rubber coating layer is highly uneven, with a rather chaotic shape and size of the distribution area. In contrast, the epoxy resin sample displays a uniform thickness distribution, with a gradual decrease in thickness from left to right. The imaging results of the two samples are different due to the preparation processes of the coating materials. The thickness characterization values are extracted as shown in Figure 16, where the maximum, minimum, average, and median thickness of the rubber coating layer is 1487.2 μm, 971.1 μm, 1262.0 μm, and 1265.1 μm, respectively. For the epoxy resin coating layer, the maximum, minimum, average, and median thickness is 1596.4 μm, 1239.2 μm, 1459.9 μm, and 1476.3 μm, respectively. Meanwhile, the variance and standard deviation of the rubber coating layer thickness are 19,845.9 and 140.9, respectively, while those of the epoxy resin are 8830.9 and 94.0. These data indicate that the thickness distribution uniformity of the epoxy resin steel plate sample is slightly superior to that of the rubber steel plate sample. In summary, the data processing model of different coating materials to terahertz spectral signals can be used to calculate the thickness of the coating layer, achieve 2D imaging thickness characterization, and extract the characteristic values of the thickness of the coating layer, such as the average, median, variance, standard deviation, and maximum and minimum values. 

**Figure 17:**
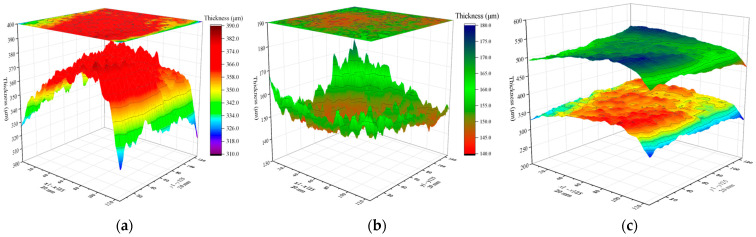
Thickness imaging of white–black sample. (**a**) White paint layer. (**b**) Black paint layer. (**c**) Double layer imaging.

**Figure 18:**
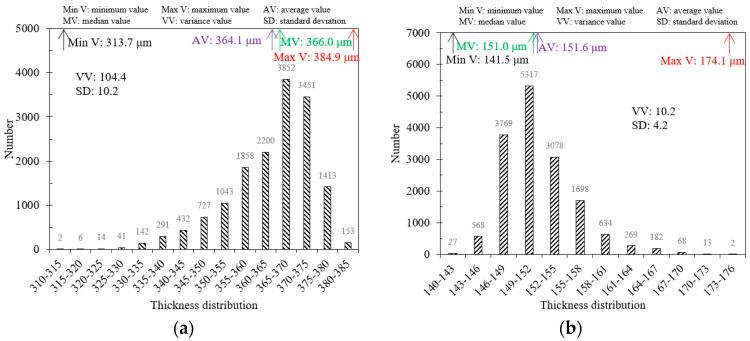
Extraction of the thickness characteristic values in (**a**) white paint layer, and (**b**) black paint layer.

In practical engineering, steel plates usually exist with different coating layer materials. Therefore, this paper uses a BP neural network model to characterize the thickness of a composite paint layer (white primer and black topcoat) on a steel plate. The scanning area size of the composite steel plate sample is 20 mm×20 mm, with a scanning resolution of x×y=125×125, resulting in 15,625 points in the scanning area. Figure 17 reveals the thickness imaging results of composite paint using the BP neural network models for white and black paints. Figure 17a–c depicts the 3D imaging results of white primer, black topcoat, and composite paint layers in the scanning area. Additionally, the characteristic values of the paint layer thickness are depicted in Figure 18. The maximum, minimum, average, and median thickness of the white primer layer is 384.9 μm, 313.7 μm, 364.1 μm, and 366.0 μm. For the black topcoat layer, the maximum, minimum, average, and median thickness is 174.1 μm, 141.5 μm, 151.6 μm, and 151.0 μm. Furthermore, the thickness of the white paint layer is mainly distributed between 355 and 375 μm, accounting for 72.7% of the total number. In contrast, the thickness distribution of the black paint layer is between 146 and 158 μm, accounting for 88.7%. By comparing the variance and standard deviation of the two layers, it can be observed that the black topcoat paint is more uniform. The experimental results demonstrate that the BP neural network model can effectively characterize the thickness of composite multi-layer coating layers, expanding the applicability range and validating the engineering significance of this model. 

### 5.2. Defects Characterization of Coating Materials

**Figure 19:**
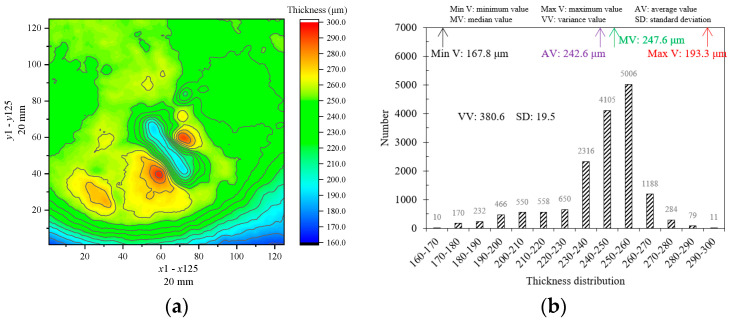
Thickness characterization of the white paint defect sample. (**a**) 2D imaging. (**b**) Thickness characteristic values.

This study employs the BP neural network model to train the terahertz signals of the steel plate sample with different coating materials, including white paint, black paint, rubber, and epoxy resin. The functional relationship between the feature parameters extracted from the terahertz time-domain signal and the coating thickness is established, enabling the thickness characterization of the coating layer in the scanning area. Furthermore, the feasibility of using this approach to identify internal coating defects is explored based on the coating thickness distribution. For the white paint steel plate sample with scratch defects, a scanning area size of 20 mm×20 mm is set, with a scanning resolution of x×y=125×125, resulting in a total of 15,625 points. The processed data from the BP neural network model are used to image the coating defect results (Figure 19). The location, size, and thickness of the scratch defect in the sample can be identified, and the thickness of the scratch defect is lower than the surrounding thickness. Additionally, two maximum thickness peaks can be identified on both sides of the scratch. The results indicate that the BP neural network model can pinpoint regions where thickness abnormalities are present across the scanning area and achieve visual thickness imaging. Further, Figure 19b displays the maximum, minimum, average, and median thickness of the defective white paint sample being 193.3 μm, 167.8 μm, 242.6 μm, and 247.6 μm, with a variance of 380.6 and a standard deviation of 19.5. The experimental results prove the capability of the proposed model in precisely identifying and extracting locational data pertaining to abnormal thickness variations within the coating layer. 

**Figure 20:**
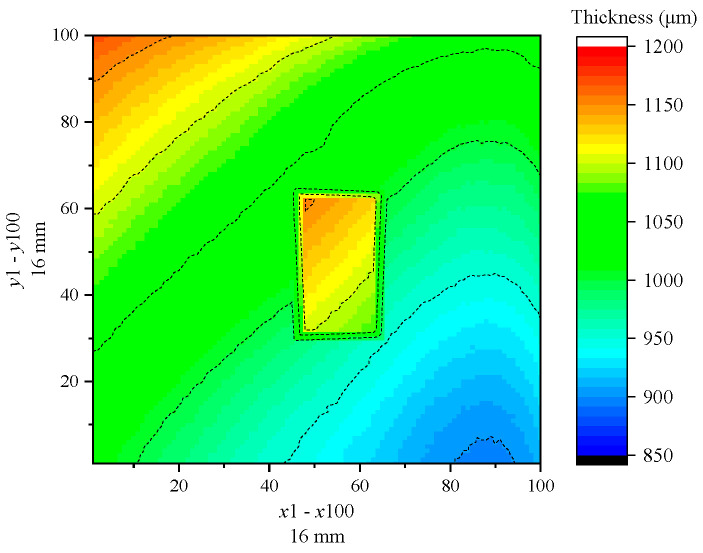
Thickness imaging of epoxy resin defect sample.

Similarly, the BP neural network model is employed to identify foreign defects in epoxy resin steel plate samples, with Figure 20 presenting the scanning area imaging results. The scanning area size is 16 mm×16 mm, with a scanning resolution of x×y=100×100, and a total of 10,000 points in the scanning area. The imaging results of the epoxy resin sample reveal a rectangular foreign defect of size 4 mm×2.5 mm, comparable to the paper used during sample preparation. The thickness distribution of epoxy resin elsewhere in the scanning area appeared more uniform. Besides, the experimental results prove that the proposed BP neural network characterizes the coating layer in the scanning area and recognizes the shape and position of the defect. It should be noted that since the disk material is not trained using the proposed BP neural network, the thickness value at the location of the foreign defect is inaccurate. The accurate detection of defects using this model is primarily contingent upon the identification of thickness abnormalities observed in the imaging outcomes, particularly abrupt thickening or thinning of materials. For a meticulous classification of the defect types, it is imperative to undertake a more comprehensive analysis, focusing on the distinctive characteristics exhibited by the terahertz time-domain reflection signals associated with various defect categories. Totally speaking, this model demonstrates its potential applicability in assessing the construction quality of coating materials for the coating layer in engineering, thereby offering a valuable tool for the non-destructive testing field. 

## 6. Conclusions

This study employs terahertz time-domain spectroscopy in conjunction with the BP neural network algorithm and proposes an effective method for thickness characterization of steel plate coating materials. The developed scheme leverages the advantages of both techniques, capitalizing on the high-resolution, real-time, and contactless measurement capabilities of terahertz time-domain spectroscopy while harnessing the predictive power of the BP neural network algorithm. The major conclusions of this work are summarized as follows:

1. A data processing model based on the BP neural network algorithm effectively fits the relationship between the coating thickness and the feature parameters extracted from the terahertz time-domain signal of coating materials. The feature parameters involve the second amplitude ρ and the delay time difference Δt. Additionally, the coating materials include white paint, black paint, epoxy resin, and rubber. Overall, the proposed model successfully maps the input parameters and the corresponding coating layer thickness with an accuracy that exceeds 96%, and further provides a novel way to obtain the refractive index of the coating materials.
2. The trained BP neural network model overcomes the dependence on refractive index values in traditional thickness calculation methods, unifies the processing process of terahertz signals for different types and thicknesses of coating materials, and provides a novel, accurate thickness characterization method for the coating layer. The model can realize 2D thickness imaging of the coating layer and extract thickness characteristic values, including the mean, median, variance, standard deviation, maximum, and minimum values.
3. The BP neural network model can identify thickness abnormalities caused by internal defects within the coating materials. Analyzing the thickness values obtained from the model makes it possible to accurately locate the position, area, and size of potential defects such as scratches and foreign object defects. The accurate detection of defects is primarily contingent upon the identification of thickness abnormalities across the scanning area.
4. The developed BP neural network model is a powerful tool for the non-destructive testing and evaluation of coating materials. It provides a reliable and efficient approach to assess the quality and integrity of coatings, identifying potential defects before they lead to performance issues or failure. This work’s findings suggest that terahertz time-domain spectroscopy combined with BP neural networks has great potential in several practical applications, including but not limited to coating material quality control, material science research, and process monitoring in manufacturing industries. Hence, this method can provide valuable insights into material properties and defects, enabling better decision-making and process control.

## Figures and Tables

**Table 1:** Parameter information of different coating materials based on BP neural network.

Materials	Black Paint	White Paint	Rubber	Epoxy Resin
**Training thickness accuracy**	98.6%	99.1%	98.9%	98.6%
**Testing thickness accuracy**	96.5%	97.1%	97.7%	97.2%
**Calculated refractive index value**	2.21	1.97	2.19	1.92

## Data Availability

The data presented in this study are available on request from the corresponding author.
